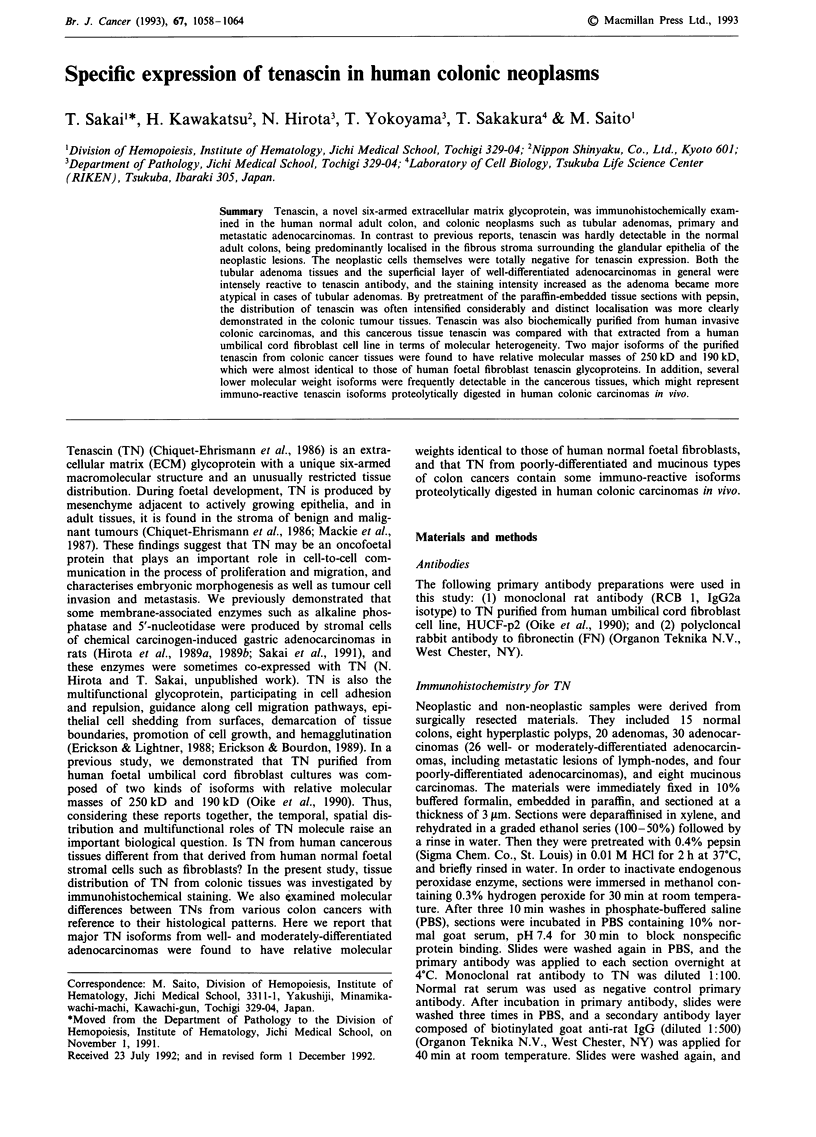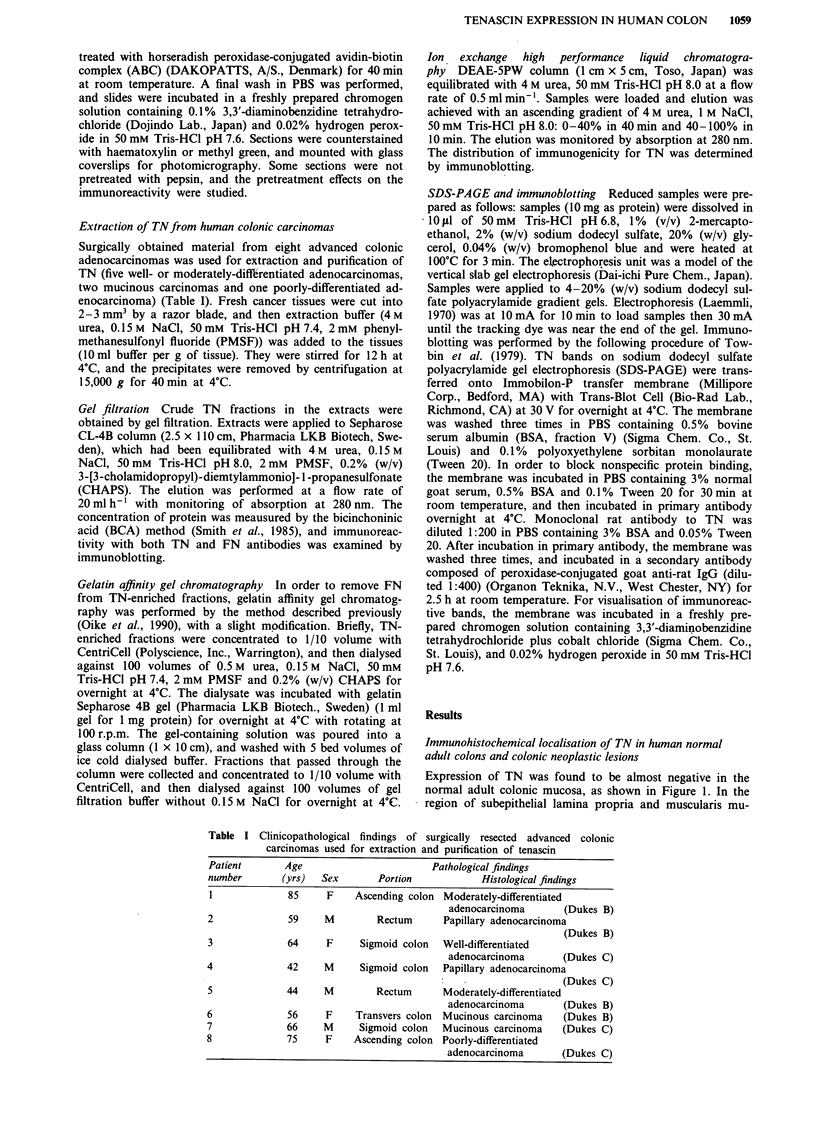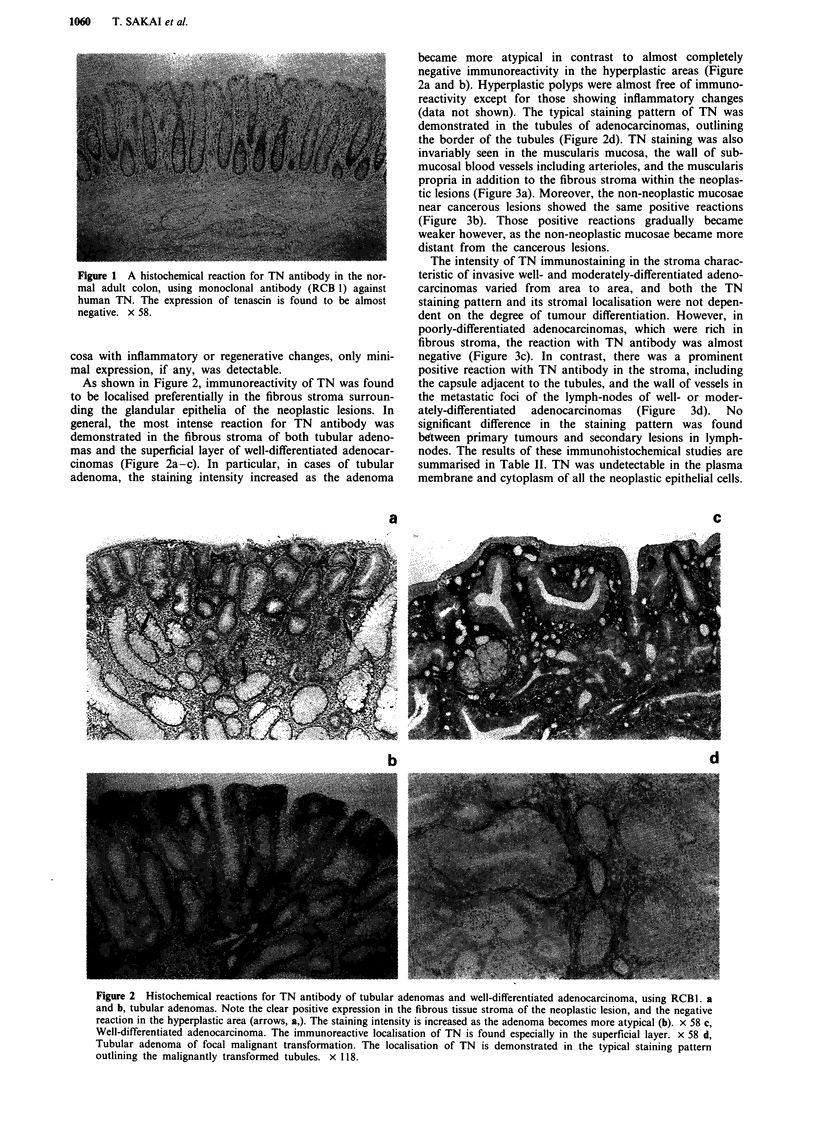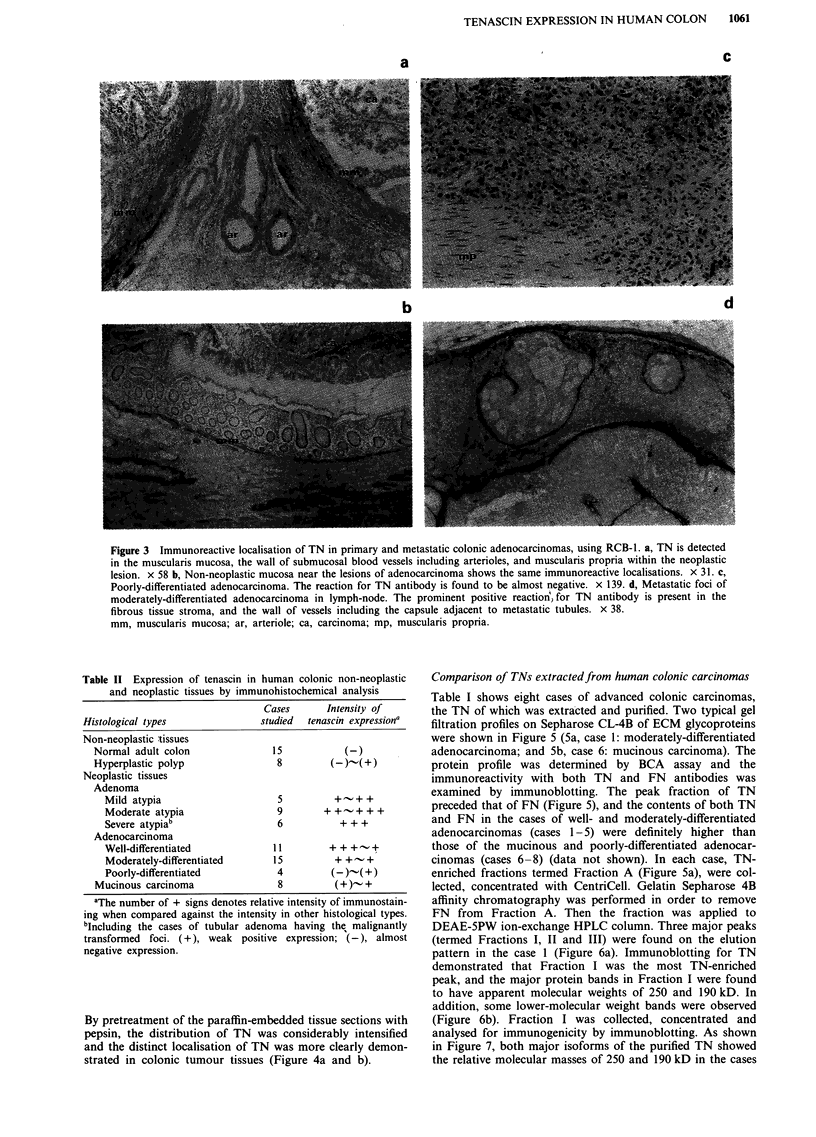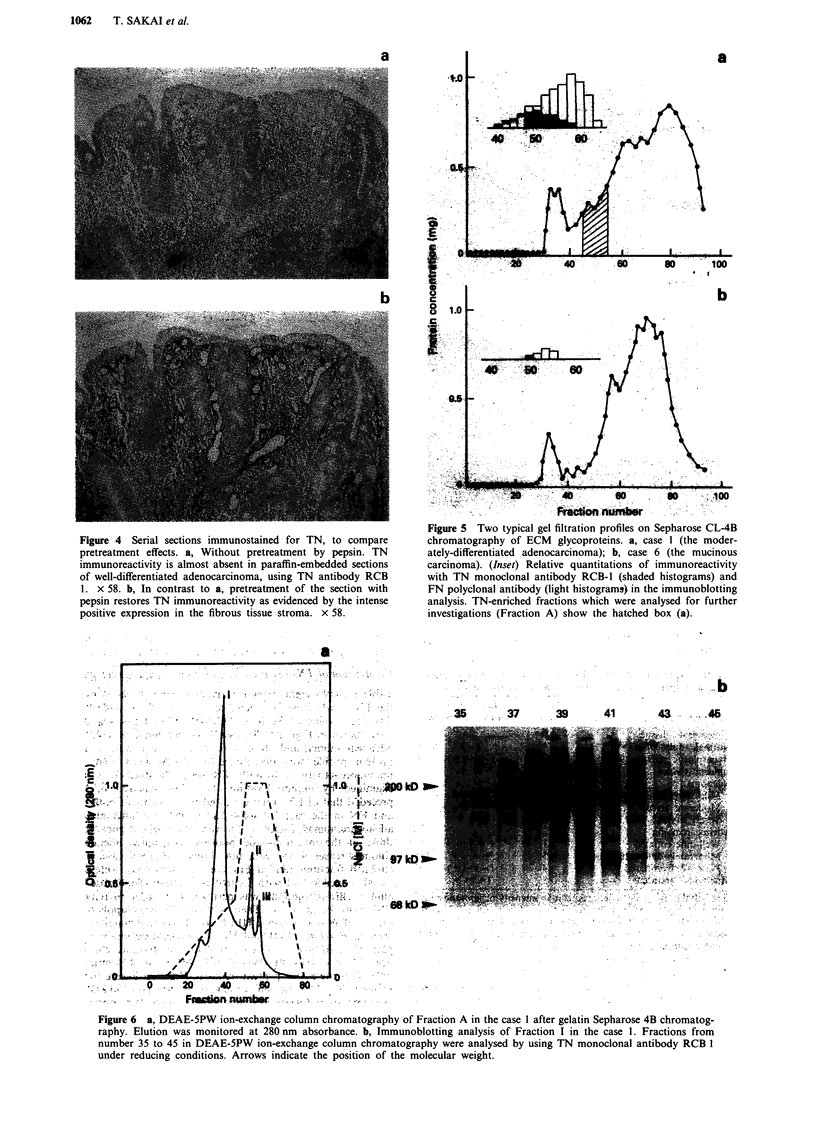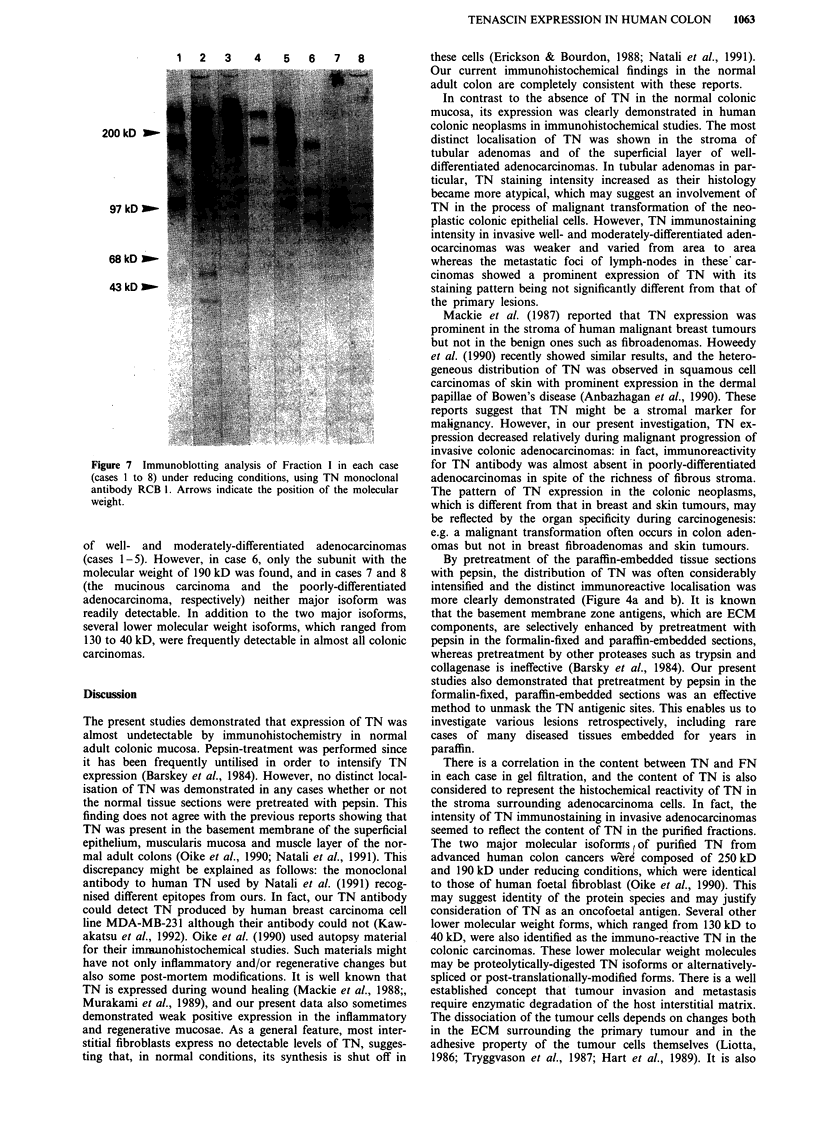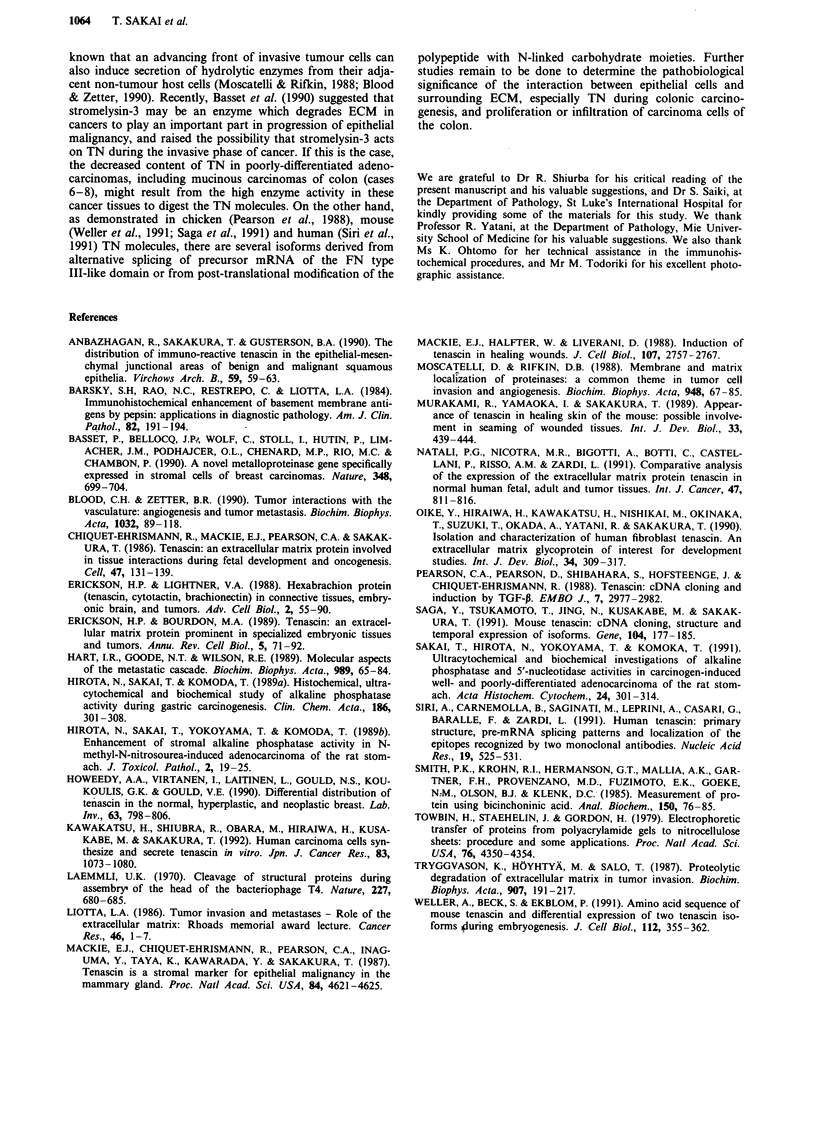# Specific expression of tenascin in human colonic neoplasms.

**DOI:** 10.1038/bjc.1993.194

**Published:** 1993-05

**Authors:** T. Sakai, H. Kawakatsu, N. Hirota, T. Yokoyama, T. Sakakura, M. Saito

**Affiliations:** Division of Hemopoiesis, Jichi Medical School, Tochigi, Japan.

## Abstract

**Images:**


Br. J. Cancer (1993), 67, 1058-1064 © Macmillan Press Ltd., 1993

### Specific expression of tenascin in human colonic neoplasms

T. Sakai[1]*, H. Kawakatsu[2], N. Hirota[3], T. Yokoyama[3], T. Sakakura[4] & M. Saito[1]

[1]*Division of Hemopoiesis, Institute of Hematology, Jichi Medical School, Tochigi 329-04;* [2]*Nippon Shinyaku, Co., Ltd., Kyoto 601;* [3]*Department of Pathology, Jichi Medical School, Tochigi 329-04;* [4]*Laboratory of Cell Biology, Tsukuba Life Science Center (RIKEN), Tsukuba, Ibaraki 305, Japan.*

**Summary** Tenascin, a novel six-armed extracellular matrix glycoprotein, was immunohistochemically examined in the human normal adult colon, and colonic neoplasms such as tubular adenomas, primary and metastatic adenocarcinomas. In contrast to previous reports, tenascin was hardly detectable in the normal adult colons, being predominantly localised in the fibrous stroma surrounding the glandular epithelia of the neoplastic lesions. The neoplastic cells themselves were totally negative for tenascin expression. Both the tubular adenoma tissues and the superficial layer of well-differentiated adenocarcinomas in general were intensely reactive to tenascin antibody, and the staining intensity increased as the adenoma became more atypical in cases of tubular adenomas. By pretreatment of the paraffin-embedded tissue sections with pepsin, the distribution of tenascin was often intensified considerably and distinct localisation was more clearly demonstrated in the colonic tumour tissues. Tenascin was also biochemically purified from human invasive colonic carcinomas, and this cancerous tissue tenascin was compared with that extracted from a human umbilical cord fibroblast cell line in terms of molecular heterogeneity. Two major isoforms of the purified tenascin from colonic cancer tissues were found to have relative molecular masses of 250 kD and 190 kD, which were almost identical to those of human foetal fibroblast tenascin glycoproteins. In addition, several lower molecular weight isoforms were frequently detectable in the cancerous tissues, which might represent immuno-reactive tenascin isoforms proteolytically digested in human colonic carcinomas *in vivo*.

Tenascin (TN) (Chiquet-Ehrismann *et al.*, 1986) is an extracellular matrix (ECM) glycoprotein with a unique six-armed macromolecular structure and an unusually restricted tissue distribution. During foetal development, TN is produced by mesenchyme adjacent to actively growing epithelia, and in adult tissues, it is found in the stroma of benign and malignant tumours (Chiquet-Ehrismann *et al.*, 1986; Mackie *et al.*, 1987). These findings suggest that TN may be an oncofoetal protein that plays an important role in cell-to-cell communication in the process of proliferation and migration, and characterises embryonic morphogenesis as well as tumour cell invasion and metastasis. We previously demonstrated that some membrane-associated enzymes such as alkaline phosphatase and 5′-nucleotidase were produced by stromal cells of chemical carcinogen-induced gastric adenocarcinomas in rats (Hirota *et al.*, 1989*a*, 1989*b*; Sakai *et al.*, 1991), and these enzymes were sometimes co-expressed with TN (N. Hirota and T. Sakai, unpublished work). TN is also the multifunctional glycoprotein, participating in cell adhesion and repulsion, guidance along cell migration pathways, epithelial cell shedding from surfaces, demarcation of tissue boundaries, promotion of cell growth, and hemagglutination (Erickson & Lightner, 1988; Erickson & Bourdon, 1989). In a previous study, we demonstrated that TN purified from human foetal umbilical cord fibroblast cultures was composed of two kinds of isoforms with relative molecular masses of 250 kD and 190 kD (Oike *et al.*, 1990). Thus, considering these reports together, the temporal, spatial distribution and multifunctional roles of TN molecule raise an important biological question. Is TN from human cancerous tissues different from that derived from human normal foetal stromal cells such as fibroblasts? In the present study, tissue distribution of TN from colonic tissues was investigated by immunohistochemical staining. We also examined molecular differences between TNs from various colon cancers with reference to their histological patterns. Here we report that major TN isoforms from well- and moderately-differentiated adenocarcinomas were found to have relative molecular weights identical to those of human normal foetal fibroblasts, and that TN from poorly-differentiated and mucinous types of colon cancers contain some immuno-reactive isoforms proteolytically digested in human colonic carcinomas *in vivo*.

### Materials and methods

### *Antibodies*

The following primary antibody preparations were used in this study: (1) monoclonal rat antibody (RCB 1, IgG2a isotype) to TN purified from human umbilical cord fibroblast cell line, HUCF-p2 (Oike *et al.*, 1990); and (2) polycloncal rabbit antibody to fibronectin (FN) (Organon Teknika N.V., West Chester, NY).

### *Immunohistochemistry for TN*

Neoplastic and non-neoplastic samples were derived from surgically resected materials. They included 15 normal colons, eight hyperplastic polyps, 20 adenomas, 30 adenocarcinomas (26 well- or moderately-differentiated adenocarcinomas, including metastatic lesions of lymph-nodes, and four poorly-differentiated adenocarcinomas), and eight mucinous carcinomas. The materials were immediately fixed in 10% buffered formalin, embedded in paraffin, and sectioned at a thickness of 3 μm. Sections were deparaffinised in xylene, and rehydrated in a graded ethanol series (100–50%) followed by a rinse in water. Then they were pretreated with 0.4% pepsin (Sigma Chem. Co., St. Louis) in 0.01 M HCl for 2 h at 37°C, and briefly rinsed in water. In order to inactivate endogenous peroxidase enzyme, sections were immersed in methanol containing 0.3% hydrogen peroxide for 30 min at room temperature. After three 10 min washes in phosphate-buffered saline (PBS), sections were incubated in PBS containing 10% normal goat serum, pH 7.4 for 30 min to block nonspecific protein binding. Slides were washed again in PBS, and the primary antibody was applied to each section overnight at 4°C. Monoclonal rat antibody to TN was diluted 1:100. Normal rat serum was used as negative control primary antibody. After incubation in primary antibody, slides were washed three times in PBS, and a secondary antibody layer composed of biotinylated goat anti-rat IgG (diluted 1:500) (Organon Teknika N.V., West Chester, NY) was applied for 40 min at room temperature. Slides were washed again, and

Correspondence: M. Saito, Division of Hemopoiesis, Institute of Hematology, Jichi Medical School, 3311-1, Yakushiji, Minamikawachi-machi, Kawachi-gun, Tochigi 329-04, Japan.
*Moved from the Department of Pathology to the Division of Hemopoiesis, Institute of Hematology, Jichi Medical School, on November 1, 1991.
Received 23 July 1992; and in revised form 1 December 1992.

treated with horseradish peroxidase-conjugated avidin-biotin complex (ABC) (DAKOPATTS, A/S., Denmark) for 40 min at room temperature. A final wash in PBS was performed, and slides were incubated in a freshly prepared chromogen solution containing 0.1% 3,3'-diaminobenzidine tetrahydrochloride (Dojindo Lab., Japan) and 0.02% hydrogen peroxide in 50 mM Tris-HCl pH 7.6. Sections were counterstained with haematoxylin or methyl green, and mounted with glass coverslips for photomicrography. Some sections were not pretreated with pepsin, and the pretreatment effects on the immunoreactivity were studied.

*Extraction of TN from human colonic carcinomas*

Surgically obtained material from eight advanced colonic adenocarcinomas was used for extraction and purification of TN (five well- or moderately-differentiated adenocarcinomas, two mucinous carcinomas and one poorly-differentiated adenocarcinoma) (Table I). Fresh cancer tissues were cut into 2–3 $mm^3$ by a razor blade, and then extraction buffer (4 M urea, 0.15 M NaCl, 50 mM Tris-HCl pH 7.4, 2 mM phenylmethanesulfonyl fluoride (PMSF)) was added to the tissues (10 ml buffer per g of tissue). They were stirred for 12 h at 4°C, and the precipitates were removed by centrifugation at 15,000 *g* for 40 min at 4°C.

*Gel filtration* Crude TN fractions in the extracts were obtained by gel filtration. Extracts were applied to Sepharose CL-4B column (2.5 × 110 cm, Pharmacia LKB Biotech, Sweden), which had been equilibrated with 4 M urea, 0.15 M NaCl, 50 mM Tris-HCl pH 8.0, 2 mM PMSF, 0.2% (w/v) 3-[3-cholamidopropyl)-diemtylammonio]-1-propanesulfonate (CHAPS). The elution was performed at a flow rate of 20 ml $h^{-1}$ with monitoring of absorption at 280 nm. The concentration of protein was meausured by the bicinchoninic acid (BCA) method (Smith *et al.*, 1985), and immunoreactivity with both TN and FN antibodies was examined by immunoblotting.

*Gelatin affinity gel chromatography* In order to remove FN from TN-enriched fractions, gelatin affinity gel chromatography was performed by the method described previously (Oike *et al.*, 1990), with a slight modification. Briefly, TN-enriched fractions were concentrated to 1/10 volume with CentriCell (Polyscience, Inc., Warrington), and then dialysed against 100 volumes of 0.5 M urea, 0.15 M NaCl, 50 mM Tris-HCl pH 7.4, 2 mM PMSF and 0.2% (w/v) CHAPS for overnight at 4°C. The dialysate was incubated with gelatin Sepharose 4B gel (Pharmacia LKB Biotech., Sweden) (1 ml gel for 1 mg protein) for overnight at 4°C with rotating at 100 r.p.m. The gel-containing solution was poured into a glass column (1 × 10 cm), and washed with 5 bed volumes of ice cold dialysed buffer. Fractions that passed through the column were collected and concentrated to 1/10 volume with CentriCell, and then dialysed against 100 volumes of gel filtration buffer without 0.15 M NaCl for overnight at 4°C.

*Ion exchange high performance liquid chromatography* DEAE-5PW column (1 cm × 5 cm, Toso, Japan) was equilibrated with 4 M urea, 50 mM Tris-HCl pH 8.0 at a flow rate of 0.5 ml $min^{-1}$. Samples were loaded and elution was achieved with an ascending gradient of 4 M urea, 1 M NaCl, 50 mM Tris-HCl pH 8.0: 0–40% in 40 min and 40–100% in 10 min. The elution was monitored by absorption at 280 nm. The distribution of immunogenicity for TN was determined by immunoblotting.

*SDS-PAGE and immunoblotting* Reduced samples were prepared as follows: samples (10 mg as protein) were dissolved in 10 µl of 50 mM Tris-HCl pH 6.8, 1% (v/v) 2-mercaptoethanol, 2% (w/v) sodium dodecyl sulfate, 20% (w/v) glycerol, 0.04% (w/v) bromophenol blue and were heated at 100°C for 3 min. The electrophoresis unit was a model of the vertical slab gel electrophoresis (Dai-ichi Pure Chem., Japan). Samples were applied to 4–20% (w/v) sodium dodecyl sulfate polyacrylamide gradient gels. Electrophoresis (Laemmli, 1970) was at 10 mA for 10 min to load samples then 30 mA until the tracking dye was near the end of the gel. Immunoblotting was performed by the following procedure of Towbin *et al.* (1979). TN bands on sodium dodecyl sulfate polyacrylamide gel electrophoresis (SDS-PAGE) were transferred onto Immobilon-P transfer membrane (Millipore Corp., Bedford, MA) with Trans-Blot Cell (Bio-Rad Lab., Richmond, CA) at 30 V for overnight at 4°C. The membrane was washed three times in PBS containing 0.5% bovine serum albumin (BSA, fraction V) (Sigma Chem. Co., St. Louis) and 0.1% polyoxyethylene sorbitan monolaurate (Tween 20). In order to block nonspecific protein binding, the membrane was incubated in PBS containing 3% normal goat serum, 0.5% BSA and 0.1% Tween 20 for 30 min at room temperature, and then incubated in primary antibody overnight at 4°C. Monoclonal rat antibody to TN was diluted 1:200 in PBS containing 3% BSA and 0.05% Tween 20. After incubation in primary antibody, the membrane was washed three times, and incubated in a secondary antibody composed of peroxidase-conjugated goat anti-rat IgG (diluted 1:400) (Organon Teknika, N.V., West Chester, NY) for 2.5 h at room temperature. For visualisation of immunoreactive bands, the membrane was incubated in a freshly prepared chromogen solution containing 3,3'-diaminobenzidine tetrahydrochloride plus cobalt chloride (Sigma Chem. Co., St. Louis), and 0.02% hydrogen peroxide in 50 mM Tris-HCl pH 7.6.

### Results

*Immunohistochemical localisation of TN in human normal adult colons and colonic neoplastic lesions*

Expression of TN was found to be almost negative in the normal adult colonic mucosa, as shown in Figure 1. In the region of subepithelial lamina propria and muscularis mu-

**Table I** Clinicopathological findings of surgically resected advanced colonic carcinomas used for extraction and purification of tenascin

| *Patient number* | *Age (yrs)* | *Sex* | *Pathological findings* *Portion* | *Histological findings* |
|---|---|---|---|---|
| 1 | 85 | F | Ascending colon | Moderately-differentiated adenocarcinoma (Dukes B) |
| 2 | 59 | M | Rectum | Papillary adenocarcinoma (Dukes B) |
| 3 | 64 | F | Sigmoid colon | Well-differentiated adenocarcinoma (Dukes C) |
| 4 | 42 | M | Sigmoid colon | Papillary adenocarcinoma (Dukes C) |
| 5 | 44 | M | Rectum | Moderately-differentiated adenocarcinoma (Dukes B) |
| 6 | 56 | F | Transvers colon | Mucinous carcinoma (Dukes B) |
| 7 | 66 | M | Sigmoid colon | Mucinous carcinoma (Dukes C) |
| 8 | 75 | F | Ascending colon | Poorly-differentiated adenocarcinoma (Dukes C) |

**Figure 1** A histochemical reaction for TN antibody in the normal adult colon, using monoclonal antibody (RCB 1) against human TN. The expression of tenascin is found to be almost negative. × 58.

cosa with inflammatory or regenerative changes, only minimal expression, if any, was detectable.

As shown in Figure 2, immunoreactivity of TN was found to be localised preferentially in the fibrous stroma surrounding the glandular epithelia of the neoplastic lesions. In general, the most intense reaction for TN antibody was demonstrated in the fibrous stroma of both tubular adenomas and the superficial layer of well-differentiated adenocarcinomas (Figure 2a–c). In particular, in cases of tubular adenoma, the staining intensity increased as the adenoma became more atypical in contrast to almost completely negative immunoreactivity in the hyperplastic areas (Figure 2a and b). Hyperplastic polyps were almost free of immunoreactivity except for those showing inflammatory changes (data not shown). The typical staining pattern of TN was demonstrated in the tubules of adenocarcinomas, outlining the border of the tubules (Figure 2d). TN staining was also invariably seen in the muscularis mucosa, the wall of submucosal blood vessels including arterioles, and the muscularis propria in addition to the fibrous stroma within the neoplastic lesions (Figure 3a). Moreover, the non-neoplastic mucosae near cancerous lesions showed the same positive reactions (Figure 3b). Those positive reactions gradually became weaker however, as the non-neoplastic mucosae became more distant from the cancerous lesions.

The intensity of TN immunostaining in the stroma characteristic of invasive well- and moderately-differentiated adenocarcinomas varied from area to area, and both the TN staining pattern and its stromal localisation were not dependent on the degree of tumour differentiation. However, in poorly-differentiated adenocarcinomas, which were rich in fibrous stroma, the reaction with TN antibody was almost negative (Figure 3c). In contrast, there was a prominent positive reaction with TN antibody in the stroma, including the capsule adjacent to the tubules, and the wall of vessels in the metastatic foci of the lymph-nodes of well- or moderately-differentiated adenocarcinomas (Figure 3d). No significant difference in the staining pattern was found between primary tumours and secondary lesions in lymph-nodes. The results of these immunohistochemical studies are summarised in Table II. TN was undetectable in the plasma membrane and cytoplasm of all the neoplastic epithelial cells.

**Figure 2** Histochemical reactions for TN antibody of tubular adenomas and well-differentiated adenocarcinoma, using RCB1. **a** and **b**, tubular adenomas. Note the clear positive expression in the fibrous tissue stroma of the neoplastic lesion, and the negative reaction in the hyperplastic area (arrows, **a**,). The staining intensity is increased as the adenoma becomes more atypical (**b**). × 58 **c**, Well-differentiated adenocarcinoma. The immunoreactive localisation of TN is found especially in the superficial layer. × 58 **d**, Tubular adenoma of focal malignant transformation. The localisation of TN is demonstrated in the typical staining pattern outlining the malignantly transformed tubules. × 118.

**Figure 3** Immunoreactive localisation of TN in primary and metastatic colonic adenocarcinomas, using RCB-1. **a**, TN is detected in the muscularis mucosa, the wall of submucosal blood vessels including arterioles, and muscularis propria within the neoplastic lesion. × 58 **b**, Non-neoplastic mucosa near the lesions of adenocarcinoma shows the same immunoreactive localisations. × 31. **c**, Poorly-differentiated adenocarcinoma. The reaction for TN antibody is found to be almost negative. × 139. **d**, Metastatic foci of moderately-differentiated adenocarcinoma in lymph-node. The prominent positive reaction, for TN antibody is present in the fibrous tissue stroma, and the wall of vessels including the capsule adjacent to metastatic tubules. × 38.
mm, muscularis mucosa; ar, arteriole; ca, carcinoma; mp, muscularis propria.

**Table II** Expression of tenascin in human colonic non-neoplastic and neoplastic tissues by immunohistochemical analysis

| *Histological types* | *Cases studied* | *Intensity of tenascin expression*[a] |
|---|---|---|
| Non-neoplastic tissues | | |
| Normal adult colon | 15 | (−) |
| Hyperplastic polyp | 8 | (−)~(+) |
| Neoplastic tissues | | |
| Adenoma | | |
| Mild atypia | 5 | +~++ |
| Moderate atypia | 9 | ++~+++ |
| Severe atypia[b] | 6 | +++ |
| Adenocarcinoma | | |
| Well-differentiated | 11 | +++~+ |
| Moderately-differentiated | 15 | ++~+ |
| Poorly-differentiated | 4 | (−)~(+) |
| Mucinous carcinoma | 8 | (+)~+ |

[a]The number of + signs denotes relative intensity of immunostaining when compared against the intensity in other histological types. [b]Including the cases of tubular adenoma having the malignantly transformed foci. (+), weak positive expression; (−), almost negative expression.

By pretreatment of the paraffin-embedded tissue sections with pepsin, the distribution of TN was considerably intensified and the distinct localisation of TN was more clearly demonstrated in colonic tumour tissues (Figure 4a and b).

*Comparison of TNs extracted from human colonic carcinomas*

Table I shows eight cases of advanced colonic carcinomas, the TN of which was extracted and purified. Two typical gel filtration profiles on Sepharose CL-4B of ECM glycoproteins were shown in Figure 5 (5a, case 1: moderately-differentiated adenocarcinoma; and 5b, case 6: mucinous carcinoma). The protein profile was determined by BCA assay and the immunoreactivity with both TN and FN antibodies was examined by immunoblotting. The peak fraction of TN preceded that of FN (Figure 5), and the contents of both TN and FN in the cases of well- and moderately-differentiated adenocarcinomas (cases 1–5) were definitely higher than those of the mucinous and poorly-differentiated adenocarcinomas (cases 6–8) (data not shown). In each case, TN-enriched fractions termed Fraction A (Figure 5a), were collected, concentrated with CentriCell. Gelatin Sepharose 4B affinity chromatography was performed in order to remove FN from Fraction A. Then the fraction was applied to DEAE-5PW ion-exchange HPLC column. Three major peaks (termed Fractions I, II and III) were found on the elution pattern in the case 1 (Figure 6a). Immunoblotting for TN demonstrated that Fraction I was the most TN-enriched peak, and the major protein bands in Fraction I were found to have apparent molecular weights of 250 and 190 kD. In addition, some lower-molecular weight bands were observed (Figure 6b). Fraction I was collected, concentrated and analysed for immunogenicity by immunoblotting. As shown in Figure 7, both major isoforms of the purified TN showed the relative molecular masses of 250 and 190 kD in the cases

**Figure 4** Serial sections immunostained for TN, to compare pretreatment effects. **a**, Without pretreatment by pepsin. TN immunoreactivity is almost absent in paraffin-embedded sections of well-differentiated adenocarcinoma, using TN antibody RCB 1. × 58. **b**, In contrast to **a**, pretreatment of the section with pepsin restores TN immunoreactivity as evidenced by the intense positive expression in the fibrous tissue stroma. × 58.

**Figure 5** Two typical gel filtration profiles on Sepharose CL-4B chromatography of ECM glycoproteins. **a**, case 1 (the moderately-differentiated adenocarcinoma); **b**, case 6 (the mucinous carcinoma). (*Inset*) Relative quantitations of immunoreactivity with TN monoclonal antibody RCB-1 (shaded histograms) and FN polyclonal antibody (light histograms) in the immunoblotting analysis. TN-enriched fractions which were analysed for further investigations (Fraction A) show the hatched box (**a**).

**Figure 6** **a**, DEAE-5PW ion-exchange column chromatography of Fraction A in the case 1 after gelatin Sepharose 4B chromatography. Elution was monitored at 280 nm absorbance. **b**, Immunoblotting analysis of Fraction I in the case 1. Fractions from number 35 to 45 in DEAE-5PW ion-exchange column chromatography were analysed by using TN monoclonal antibody RCB 1 under reducing conditions. Arrows indicate the position of the molecular weight.

**Figure 7** Immunoblotting analysis of Fraction I in each case (cases 1 to 8) under reducing conditions, using TN monoclonal antibody RCB 1. Arrows indicate the position of the molecular weight.

of well- and moderately-differentiated adenocarcinomas (cases 1–5). However, in case 6, only the subunit with the molecular weight of 190 kD was found, and in cases 7 and 8 (the mucinous carcinoma and the poorly-differentiated adenocarcinoma, respectively) neither major isoform was readily detectable. In addition to the two major isoforms, several lower molecular weight isoforms, which ranged from 130 to 40 kD, were frequently detectable in almost all colonic carcinomas.

### Discussion

The present studies demonstrated that expression of TN was almost undetectable by immunohistochemistry in normal adult colonic mucosa. Pepsin-treatment was performed since it has been frequently untilised in order to intensify TN expression (Barskey *et al.*, 1984). However, no distinct localisation of TN was demonstrated in any cases whether or not the normal tissue sections were pretreated with pepsin. This finding does not agree with the previous reports showing that TN was present in the basement membrane of the superficial epithelium, muscularis mucosa and muscle layer of the normal adult colons (Oike *et al.*, 1990; Natali *et al.*, 1991). This discrepancy might be explained as follows: the monoclonal antibody to human TN used by Natali *et al.* (1991) recognised different epitopes from ours. In fact, our TN antibody could detect TN produced by human breast carcinoma cell line MDA-MB-231 although their antibody could not (Kawakatsu *et al.*, 1992). Oike *et al.* (1990) used autopsy material for their immunohistochemical studies. Such materials might have not only inflammatory and/or regenerative changes but also some post-mortem modifications. It is well known that TN is expressed during wound healing (Mackie *et al.*, 1988;, Murakami *et al.*, 1989), and our present data also sometimes demonstrated weak positive expression in the inflammatory and regenerative mucosae. As a general feature, most interstitial fibroblasts express no detectable levels of TN, suggesting that, in normal conditions, its synthesis is shut off in these cells (Erickson & Bourdon, 1988; Natali *et al.*, 1991). Our current immunohistochemical findings in the normal adult colon are completely consistent with these reports.

In contrast to the absence of TN in the normal colonic mucosa, its expression was clearly demonstrated in human colonic neoplasms in immunohistochemical studies. The most distinct localisation of TN was shown in the stroma of tubular adenomas and of the superficial layer of well-differentiated adenocarcinomas. In tubular adenomas in particular, TN staining intensity increased as their histology became more atypical, which may suggest an involvement of TN in the process of malignant transformation of the neoplastic colonic epithelial cells. However, TN immunostaining intensity in invasive well- and moderately-differentiated adenocarcinomas was weaker and varied from area to area whereas the metastatic foci of lymph-nodes in these carcinomas showed a prominent expression of TN with its staining pattern being not significantly different from that of the primary lesions.

Mackie *et al.* (1987) reported that TN expression was prominent in the stroma of human malignant breast tumours but not in the benign ones such as fibroadenomas. Howeedy *et al.* (1990) recently showed similar results, and the heterogeneous distribution of TN was observed in squamous cell carcinomas of skin with prominent expression in the dermal papillae of Bowen's disease (Anbazhagan *et al.*, 1990). These reports suggest that TN might be a stromal marker for malignancy. However, in our present investigation, TN expression decreased relatively during malignant progression of invasive colonic adenocarcinomas: in fact, immunoreactivity for TN antibody was almost absent in poorly-differentiated adenocarcinomas in spite of the richness of fibrous stroma. The pattern of TN expression in the colonic neoplasms, which is different from that in breast and skin tumours, may be reflected by the organ specificity during carcinogenesis: e.g. a malignant transformation often occurs in colon adenomas but not in breast fibroadenomas and skin tumours.

By pretreatment of the paraffin-embedded tissue sections with pepsin, the distribution of TN was often considerably intensified and the distinct immunoreactive localisation was more clearly demonstrated (Figure 4a and b). It is known that the basement membrane zone antigens, which are ECM components, are selectively enhanced by pretreatment with pepsin in the formalin-fixed and paraffin-embedded sections, whereas pretreatment by other proteases such as trypsin and collagenase is ineffective (Barsky *et al.*, 1984). Our present studies also demonstrated that pretreatment by pepsin in the formalin-fixed, paraffin-embedded sections was an effective method to unmask the TN antigenic sites. This enables us to investigate various lesions retrospectively, including rare cases of many diseased tissues embedded for years in paraffin.

There is a correlation in the content between TN and FN in each case in gel filtration, and the content of TN is also considered to represent the histochemical reactivity of TN in the stroma surrounding adenocarcinoma cells. In fact, the intensity of TN immunostaining in invasive adenocarcinomas seemed to reflect the content of TN in the purified fractions. The two major molecular isoforms of purified TN from advanced human colon cancers were composed of 250 kD and 190 kD under reducing conditions, which were identical to those of human foetal fibroblast (Oike *et al.*, 1990). This may suggest identity of the protein species and may justify consideration of TN as an oncofoetal antigen. Several other lower molecular weight forms, which ranged from 130 kD to 40 kD, were also identified as the immuno-reactive TN in the colonic carcinomas. These lower molecular weight molecules may be proteolytically-digested TN isoforms or alternatively-spliced or post-translationally-modified forms. There is a well established concept that tumour invasion and metastasis require enzymatic degradation of the host interstitial matrix. The dissociation of the tumour cells depends on changes both in the ECM surrounding the primary tumour and in the adhesive property of the tumour cells themselves (Liotta, 1986; Tryggvason *et al.*, 1987; Hart *et al.*, 1989). It is also known that an advancing front of invasive tumour cells can also induce secretion of hydrolytic enzymes from their adjacent non-tumour host cells (Moscatelli & Rifkin, 1988; Blood & Zetter, 1990). Recently, Basset *et al.* (1990) suggested that stromelysin-3 may be an enzyme which degrades ECM in cancers to play an important part in progression of epithelial malignancy, and raised the possibility that stromelysin-3 acts on TN during the invasive phase of cancer. If this is the case, the decreased content of TN in poorly-differentiated adenocarcinomas, including mucinous carcinomas of colon (cases 6–8), might result from the high enzyme activity in these cancer tissues to digest the TN molecules. On the other hand, as demonstrated in chicken (Pearson *et al.*, 1988), mouse (Weller *et al.*, 1991; Saga *et al.*, 1991) and human (Siri *et al.*, 1991) TN molecules, there are several isoforms derived from alternative splicing of precursor mRNA of the FN type III-like domain or from post-translational modification of the polypeptide with N-linked carbohydrate moieties. Further studies remain to be done to determine the pathobiological significance of the interaction between epithelial cells and surrounding ECM, especially TN during colonic carcinogenesis, and proliferation or infiltration of carcinoma cells of the colon.

We are grateful to Dr R. Shiurba for his critical reading of the present manuscript and his valuable suggestions, and Dr S. Saiki, at the Department of Pathology, St Luke's International Hospital for kindly providing some of the materials for this study. We thank Professor R. Yatani, at the Department of Pathology, Mie University School of Medicine for his valuable suggestions. We also thank Ms K. Ohtomo for her technical assistance in the immunohistochemical procedures, and Mr M. Todoriki for his excellent photographic assistance.

### References

ANBAZHAGAN, R., SAKAKURA, T. & GUSTERSON, B.A. (1990). The distribution of immuno-reactive tenascin in the epithelial-mesenchymal junctional areas of benign and malignant squamous epithelia. *Virchows Arch. B.*, **59**, 59–63.

BARSKY, S.H, RAO, N.C., RESTREPO, C. & LIOTTA, L.A. (1984). Immunohistochemical enhancement of basement membrane antigens by pepsin: applications in diagnostic pathology. *Am. J. Clin. Pathol.*, **82**, 191–194.

BASSET, P., BELLOCQ, J.P., WOLF, C., STOLL, I., HUTIN, P., LIMACHER, J.M., PODHAJCER, O.L., CHENARD, M.P., RIO, M.C. & CHAMBON, P. (1990). A novel metalloproteinase gene specifically expressed in stromal cells of breast carcinomas. *Nature*, **348**, 699–704.

BLOOD, C.H. & ZETTER, B.R. (1990). Tumor interactions with the vasculature: angiogenesis and tumor metastasis. *Biochim. Biophys. Acta*, **1032**, 89–118.

CHIQUET-EHRISMANN, R., MACKIE, E.J., PEARSON, C.A. & SAKAKURA, T. (1986). Tenascin: an extracellular matrix protein involved in tissue interactions during fetal development and oncogenesis. *Cell*, **47**, 131–139.

ERICKSON, H.P. & LIGHTNER, V.A. (1988). Hexabrachion protein (tenascin, cytotactin, brachionectin) in connective tissues, embryonic brain, and tumors. *Adv. Cell Biol.*, **2**, 55–90.

ERICKSON, H.P. & BOURDON, M.A. (1989). Tenascin: an extracellular matrix protein prominent in specialized embryonic tissues and tumors. *Annu. Rev. Cell Biol.*, **5**, 71–92.

HART, I.R., GOODE, N.T. & WILSON, R.E. (1989). Molecular aspects of the metastatic cascade. *Biochim. Biophys. Acta.*, **989**, 65–84.

HIROTA, N., SAKAI, T. & KOMODA, T. (1989*a*). Histochemical, ultracytochemical and biochemical study of alkaline phosphatase activity during gastric carcinogenesis. *Clin. Chem. Acta.*, **186**, 301–308.

HIROTA, N., SAKAI, T., YOKOYAMA, T. & KOMODA, T. (1989*b*). Enhancement of stromal alkaline phosphatase activity in N-methyl-N-nitrosourea-induced adenocarcinoma of the rat stomach. *J. Toxicol. Pathol.*, **2**, 19–25.

HOWEEDY, A.A., VIRTANEN, I., LAITINEN, L., GOULD, N.S., KOUKOULIS, G.K. & GOULD, V.E. (1990). Differential distribution of tenascin in the normal, hyperplastic, and neoplastic breast. *Lab. Inv.*, **63**, 798–806.

KAWAKATSU, H., SHIUBRA, R., OBARA, M., HIRAIWA, H., KUSAKABE, M. & SAKAKURA, T. (1992). Human carcinoma cells synthesize and secrete tenascin *in vitro*. *Jpn. J. Cancer Res.*, **83**, 1073–1080.

LAEMMLI, U.K. (1970). Cleavage of structural proteins during assembry of the head of the bacteriophage T4. *Nature*, **227**, 680–685.

LIOTTA, L.A. (1986). Tumor invasion and metastases – Role of the extracellular matrix: Rhoads memorial award lecture. *Cancer Res.*, **46**, 1–7.

MACKIE, E.J., CHIQUET-EHRISMANN, R., PEARSON, C.A., INAGUMA, Y., TAYA, K., KAWARADA, Y. & SAKAKURA, T. (1987). Tenascin is a stromal marker for epithelial malignancy in the mammary gland. *Proc. Natl Acad. Sci. USA*, **84**, 4621–4625.

MACKIE, E.J., HALFTER, W. & LIVERANI, D. (1988). Induction of tenascin in healing wounds. *J. Cell Biol.*, **107**, 2757–2767.

MOSCATELLI, D. & RIFKIN, D.B. (1988). Membrane and matrix localization of proteinases: a common theme in tumor cell invasion and angiogenesis. *Biochim. Biophys. Acta*, **948**, 67–85.

MURAKAMI, R., YAMAOKA, I. & SAKAKURA, T. (1989). Appearance of tenascin in healing skin of the mouse: possible involvement in seaming of wounded tissues. *Int. J. Dev. Biol.*, **33**, 439–444.

NATALI, P.G., NICOTRA, M.R., BIGOTTI, A., BOTTI, C., CASTELLANI, P., RISSO, A.M. & ZARDI, L. (1991). Comparative analysis of the expression of the extracellular matrix protein tenascin in normal human fetal, adult and tumor tissues. *Int. J. Cancer*, **47**, 811–816.

OIKE, Y., HIRAIWA, H., KAWAKATSU, H., NISHIKAI, M., OKINAKA, T., SUZUKI, T., OKADA, A., YATANI, R. & SAKAKURA, T. (1990). Isolation and characterization of human fibroblast tenascin. An extracellular matrix glycoprotein of interest for development studies. *Int. J. Dev. Biol.*, **34**, 309–317.

PEARSON, C.A., PEARSON, D., SHIBAHARA, S., HOFSTEENGE, J. & CHIQUET-EHRISMANN, R. (1988). Tenascin: cDNA cloning and induction by TGF-$\beta$. *EMBO J.*, **7**, 2977–2982.

SAGA, Y., TSUKAMOTO, T., JING, N., KUSAKABE, M. & SAKAKURA, T. (1991). Mouse tenascin: cDNA cloning, structure and temporal expression of isoforms. *Gene*, **104**, 177–185.

SAKAI, T., HIROTA, N., YOKOYAMA, T. & KOMOKA, T. (1991). Ultracytochemical and biochemical investigations of alkaline phosphatase and 5′-nucleotidase activities in carcinogen-induced well- and poorly-differentiated adenocarcinoma of the rat stomach. *Acta Histochem. Cytochem.*, **24**, 301–314.

SIRI, A., CARNEMOLLA, B., SAGINATI, M., LEPRINI, A., CASARI, G., BARALLE, F. & ZARDI, L. (1991). Human tenascin: primary structure, pre-mRNA splicing patterns and localization of the epitopes recognized by two monoclonal antibodies. *Nucleic Acid Res.*, **19**, 525–531.

SMITH, P.K., KROHN, R.I., HERMANSON, G.T., MALLIA, A.K., GARTNER, F.H., PROVENZANO, M.D., FUZIMOTO, E.K., GOEKE, N.M., OLSON, B.J. & KLENK, D.C. (1985). Measurement of protein using bicinchoninic acid. *Anal. Biochem.*, **150**, 76–85.

TOWBIN, H., STAEHELIN, J. & GORDON, H. (1979). Electrophoretic transfer of proteins from polyacrylamide gels to nitrocellulose sheets: procedure and some applications. *Proc. Natl Acad. Sci. USA*, **76**, 4350–4354.

TRYGGVASON, K., HÖYHTYÄ, M. & SALO, T. (1987). Proteolytic degradation of extracellular matrix in tumor invasion. *Biochim. Biophys. Acta.*, **907**, 191–217.

WELLER, A., BECK, S. & EKBLOM, P. (1991). Amino acid sequence of mouse tenascin and differential expression of two tenascin isoforms during embryogenesis. *J. Cell Biol.*, **112**, 355–362.